# Wealth-based disparities in the prevalence of short birth interval in India: insights from NFHS-5

**DOI:** 10.1186/s12963-024-00334-0

**Published:** 2024-07-11

**Authors:** Aditya Singh, Anshika Singh, Mahashweta Chakrabarty, Shivani Singh, Pooja Tripathi

**Affiliations:** 1 Department of Geography, Banaras Hindu University, Varanasi, Uttar Pradesh India; 2 Independent Researcher, Lucknow, Uttar Pradesh India; 3 Girl Innovation, Research, and Learning Center, Population Council, New York, USA

**Keywords:** Short birth interval, Wealth-based inequality, NFHS-5, Erreygers normalized concentration index, India

## Abstract

**Background:**

Short birth interval (SBI) has profound implications for the health of both mothers and children, yet there remains a notable dearth of studies addressing wealth-based inequality in SBI and its associated factors in India. This study aims to address this gap by investigating wealth-based disparities in SBI and identifying the underlying factors associated with SBI in India.

**Methods:**

We used information on 109,439 women of reproductive age (15–49 years) from the fifth round of the National Family Health Survey (2019-21). We assessed wealth-based inequality in SBI for India and its states using the Erreygers Normalised Concentration Index (ECI). Additionally, we used a multilevel binary logistic regression to assess the factors associated with SBI in India.

**Results:**

In India, the prevalence of SBI was 47.8% [95% CI: 47.4, 48.3] during 2019-21, with significant variation across states. Bihar reported the highest prevalence of SBI at 61.2%, while Sikkim the lowest at 18.1%. SBI prevalence was higher among poorer mothers compared to richer ones (Richest: 33.8% vs. Poorest: 52.9%). This wealth-based inequality was visible in the ECI as well (ECI= -0.13, *p* < 0.001). However, ECI varied considerably across the states. Gujarat, Punjab, and Manipur exhibited the highest levels of wealth-based inequality (ECI= -0.28, *p* < 0.001), whereas Kerala showed minimal wealth-based inequality (ECI= -0.01, *p* = 0.643). Multilevel logistic regression analysis identified several factors associated with SBI. Mothers aged 15–24 (OR: 12.01, *p* < 0.001) and 25–34 (2.92, < 0.001) were more likely to experience SBI. Women who married after age 25 (3.17, < 0.001) and those belonging to Scheduled Caste (1.18, < 0.001), Scheduled Tribes (1.14, < 0.001), and Other Backward Classes (1.12, < 0.001) also had higher odds of SBI. Additionally, the odds of SBI were higher among mothers in the poorest (1.97, < 0.001), poorer (1.73, < 0.001), middle (1.62, < 0.001), and richer (1.39, < 0.001) quintiles compared to the richest quintile. Women whose last child had passed away were also significantly more likely to have SBI (2.35, < 0.001). Furthermore, mothers from communities with lower average schooling levels (1.18, < 0.001) were more likely to have SBI. Geographically, mothers from eastern (0.67, < 0.001) and northeastern (0.44, < 0.001) regions of India were less likely to have SBI.

**Conclusion:**

The significant wealth-based inequality in SBI in India highlights the need for targeted interventions focusing on economically disadvantaged women, particularly in states with high SBI prevalence. Special attention should be given to younger mothers and those from socially disadvantaged groups to enhance maternal and child health outcomes across the country.

**Supplementary Information:**

The online version contains supplementary material available at 10.1186/s12963-024-00334-0.

## Introduction

Birth intervals, the duration between two consecutive births, hold significant implications for maternal and child health. World Health Organization (WHO) recommends an interval of 24 months for birth-to-pregnancy and 33 months for birth-to-birth, assuming a gestation of 9 months for a healthy pregnancy and birth [1]. A birth interval of lesser than 33 months is termed a ‘short birth interval’ (SBI). An adequate birth interval is crucial for a mother’s recovery from the physical demands of pregnancy and childbirth, adequate lactation, nutrition, and equitable distribution of resources among all children [2]. In contrast, an SBI can lead to various health issues and morbidities for both the mother and child. These may include poor nutrition, underweight and stunting, less time spent breastfeeding, infections, high blood pressure, complications for the mother, and incomplete healing of the uterus [3–6]. When birth-to-birth spacing is extremely short, it may result in serious complications such neonatal and infant death, preterm delivery, reduced birth weight, smaller-than-expected size for gestational age, early membrane rupture, uterine tearing, placental separation, abnormal placental position, and pregnancy-induced hypertension [2, 5, 7–10].

Previous research conducted in low-and-middle-income countries, including India, has examined the issue of SBI and found that women’s demographic characteristics, including age, education, age at marriage, contraceptive use, parity, survival status of previous child, gender of previous child, preference for a son, and duration of breastfeeding of the previous child, have a significant impact on birth intervals [2, 5, 6, 8–30]. Similarly, socioeconomic and geographical factors, such as social group, religion, household wealth, autonomy, mass media exposure, place of residence, and region of residence have also been found to be associated with SBI [8, 13, 22, 31]. A few studies have attempted to investigate socioeconomic inequality in the prevalence of SBI [11, 15].

Health inequality or disparity across socioeconomic groups is an unjust difference in health and this is often viewed as objectionable from a human rights perspective [32]. Socioeconomic inequalities in health-related outcomes are common in most low-and-middle-income countries [15]. Therefore, health inequalities challenges have become a priority area for global organizations like WHO [1, 33] and United Nations Population Fund [34]. As the SBI could contribute to neonatal mortality, the Goal 3 of Sustainable Development Goals established by the United Nations, specifically targets 3.2.1 and 3.2.2, aim to eradicate preventable deaths among neonates and under-5 children. Each nation strives to achieve a reduction in neonatal mortality to below 12 deaths per 1,000 live births and under-five mortality to below 25 deaths per 1,000 live births by 2030, as shorter birth intervals have been linked to higher neonatal mortality rates. Additionally, the goal 10 of the SDGs seeks to reduce inequality within and among nations [35].

There is dearth of studies exploring socioeconomic disparity in SBI in India. The previous studies carried out in India on the issue of SBI mainly investigate significant correlates of birth interval on the national level, while some others have been conducted at smaller scales [3, 6, 12, 13, 18, 20, 21, 36]. While small-scale studies offer valuable insights into health behaviours, their findings cannot be applied to larger populations due to limited geographic representation and small sample sizes, which restrict their statistical power. The studies conducted at national level have used mostly the data from the nationally representative surveys such as the fourth round of the National Family Health Survey (NFHS) with hierarchical sampling designs. However, these studies have not used appropriate regression models for such data sets. Therefore, this study aims to address these gaps by pursuing two main objectives: firstly, measuring and analysing wealth-based inequality in SBI across different states of India, and secondly, examining the factors associated with SBI using a multilevel modelling approach.

## Data and methods

### Data source

**Fig. 1:**
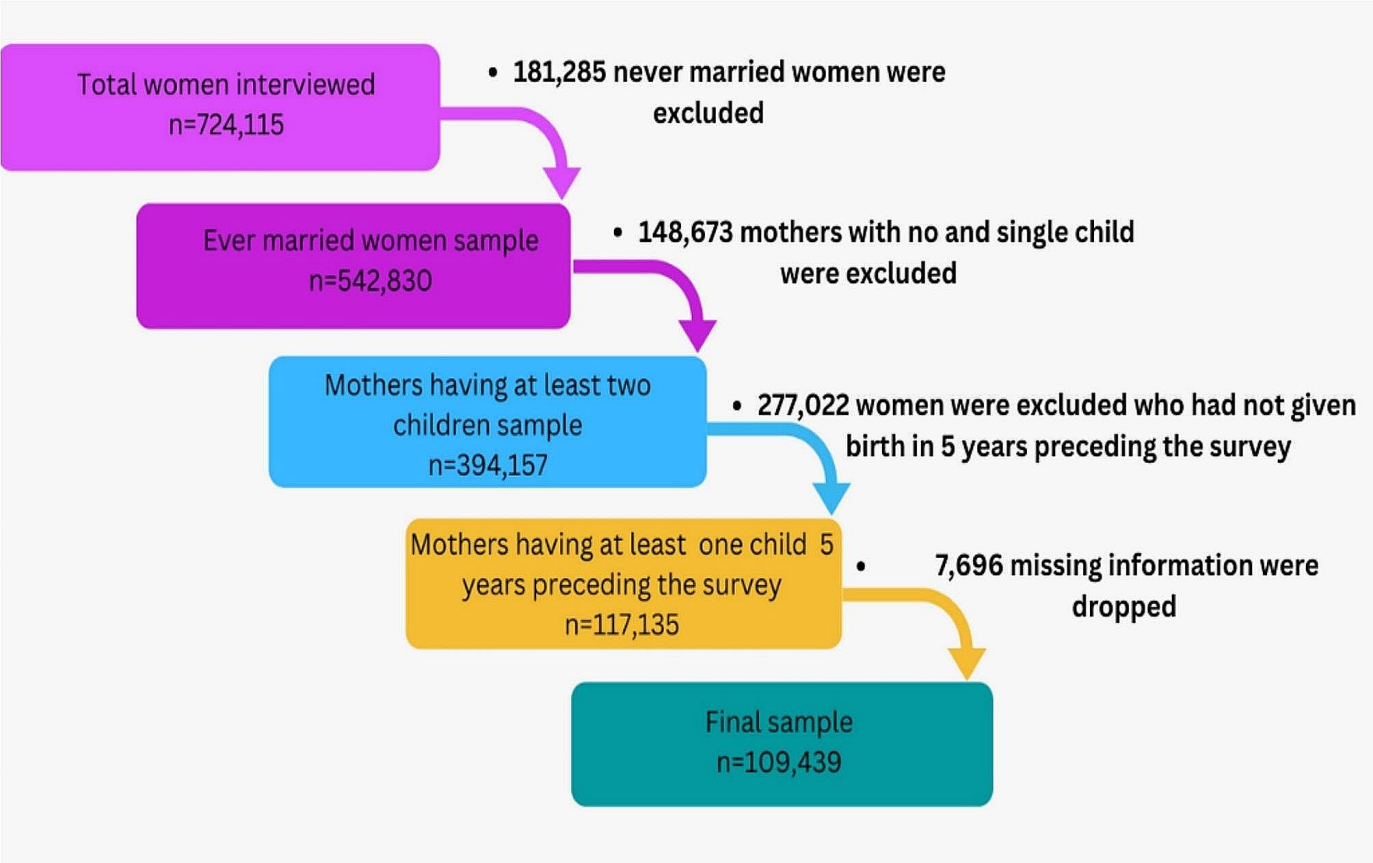
Flow chart showing the process of sample selection from the NFHS-5 dataset

We utilized data from the fifth round of the NFHS conducted during 2019-21. This survey covers a number of issues related to reproductive and child health, fertility, family planning, morbidity and mortality among Indian population. A two-stage sampling design was adopted in NFHS-5. Further details about the sampling design can be found in the NFHS-5 national reports [37]. NFHS-5 covered a sample of 724,115 women of reproductive age (15–49 years) from 636,699 households. The response rate was close to 97%. For this study, a subset of 109,439 women aged 15–49 from various Indian states and union territories (UT) was selected. The details of how we arrived at this sample of 109,439 women are provided in Fig. 1. We had to exclude 7696 observations due to missing values. Over 80% of these were excluded due to missing values in the variable ‘social group’. The rest of the excluded observation were either missing or inconsistent in variables related to age at marriage and birth history. Upon comparing the characteristics of excluded (missing) and included (non-missing) observations in our study dataset, we found only minor differences between these two groups (see Table A1 in Appendix).

### Statistical analysis

We initially calculated the prevalence of SBI for India and its states. Following this, we computed the Erreygers Normalised Concentration Index at both national and state levels to analyse wealth-based inequalities. We used the words disparity and inequality interchangably throughout the paper. Finally, multilevel logistic regression analysis was employed to identify the factors affecting SBI occurrence in India.

#### Erreygers normalised concentration index

We used Erreygers Normalised Concentration Index (ECI) to assess wealth-based inequality in SBI for India and each of its states and union territories. The ECI is modified version of the widely used Concentration Index (CI) which is a useful tool for measuring socioeconomic inequality in health sector variables. Mathematically “the concentration index is defined as twice the area between the concentration curve and the line of equality (the 45-degree line)”, which can be equated as [38]:

$$C=\frac{2}{\mu }cov\left(h,r\right)$$

Where C is the concentration index, µ is the mean of the health variable, h is the health variable, and r is the cumulative percentage. The index ranges between − 1 to + 1 for an unbounded variable and µ-1 to 1- µ for a bounded variable.

However, when the health variable is binary, the mean of the distribution determines the limits of the potential values of the concentration index: as the mean increases, the range of possible concentration index values narrows [39]. Wagstaff suggested a normalization formula to address this limitation. He proposes dividing the health concentration index by its upper bound [39, 40]. This modified Wagstaff index for binary health variables only fulfils the transfer property, mirror property, and the level of independence property, but not the cardinal invariance. To develop a socio-economic inequality index that is rank-dependent and satisfies all four properties, Erreygers has introduced a corrected concentration index (also known as Erreygers Normalised Concentration Index) as [40]:

$$E(h)= \frac{8}{{n}^{2\left({b}_{h}-{a}_{h}\right)}}\sum _{i=1}^{n}{z}_{i}{h}_{i}$$

Where the n is the sample size, b_h_ is the maximum value of the health variable, a_h_ is the minimum value of the health variable, z_i =_$\frac{n+1}{2}- \lambda$_i,_ and h_i_ is a binary health variable that is equal to 0 when the woman with no SBI and takes a value of 1 otherwise.

#### Multilevel binary logistic regression

A multilevel binary logistic regression approach was utilized to explore the association of various factors at the individual, community, and district levels on SBI. Opting for a three-level logistic regression over a simple regression approach was deliberate, as it accommodates the hierarchical structure of the NFHS-5 dataset, where individuals (mothers) are nested within communities or primary sampling units (PSUs), and communities within districts. This multilevel framework is well-suited for such nested datasets, providing standard errors (SEs) adjusted for clustering effects. Conversely, relying on SEs estimated through a simple binary logistic regression model may underestimate SEs when the dataset used is hierarchical in nature, potentially impacting the interpretation of regression outcomes.

Before applying the three-level regression, we examined the extent to which the outcome of interest varies at higher levels (community and district levels). We fitted a null model, also known as empty model, contained no independent variables. We found that a considerable proportion of the total variation in SBI was at community and district levels. This suggested fitting a multilevel model made sense in our context. In Model 2, factors at the individual, community, and district levels were collectively incorporated. The fixed effects, indicating measures of association, are presented as odds ratios (ORs) with 95% confidence intervals. Meanwhile, the random effects, indicating measures of variation, are displayed as variance partition coefficients (VPCs) [41]. To evaluate multicollinearity in the regression model, we employed the variance inflation factor (VIF). All VIF values were comfortably below 2.4, a threshold indicating concern for multicollinearity affecting results (see Appendix Table A2). We utilized Stata 16 and MLwiN to carry out the multilevel logistic regression analysis [42].

Dependent variable: The dependent variable was SBI defined as a minimum interval of 24 months between a live birth and attempting the subsequent pregnancy [1]. When accounting for the nine-month gestational period, this translates to a birth-to-birth spacing of 33 months. In the NFHS-5, women were asked about their children’s birth dates using the century month code. The variable “preceding birth interval” was created by subtracting the birth date of the last child from that of the previous child, resulting in the duration of the birth interval in months. We recoded the birth interval as “1” if its duration was less than 33 months and as “0” if it was 33 months or longer.

Independent variables: Building upon existing literature, we incorporated a range of biodemographic, socioeconomic, family planning, and geographical factors into our analysis [10, 11, 13, 15, 19, 23–25, 28, 29, 43]. These factors included the age of women, women’s education level, age at marriage, social group, religion, place of residence, exposure to mass media, number of children prior to the index child, survival status of previous children, sex of the previous child, preference for a son, level of poverty in the community, level of education in the community, level of exposure to mass media regarding family planning in the community, and region of residence. For detailed information on the coding of these variables, please refer to Table 1.

**Table 1:** Description of variables used in the study

Variables	Description
Age group	Respondent’s current age was grouped into three categories- ‘15–24 years’ (coded as 1), ‘25–34 years’ (coded as 2), and ‘35–49 years’ (coded as 3)
Age at marriage	Respondents were asked about their age in years at their first marriage. The variable was generated with three categories- ‘Less than 20 years’ (coded as 1), ’20–25 years’ (coded as 2), and ‘More than 25 years’ (coded as 3)
Level of education	Asked respondents what is their highest year of education. Those who had never been to school were labelled as ‘not educated’ (coded as 0) and those who attained primary level, secondary level and higher level of education were labelled as ‘Primary’ (coded as 1), ‘Secondary’ (coded as 2), and ‘Higher’ (coded as 3)
Social groups	There are four official social groups, i.e., ‘Scheduled Caste’ (coded as 1), ‘Scheduled Tribe’ (coded as 2), ‘Other Backward Caste’ (coded as 3) and ‘Others’ (coded as 0).
Religion	Respondent’s religion has three categories- ‘Hindu’ (coded as 0), ‘Muslim’ (coded as 01), and ‘Others’ (coded as 2) (including Christian, Sikh, Buddhist/Neo-Buddhist, Jain, Jewish, Parsi/Zoroastrian, no religion, and other)
Household wealth index	The wealth index is a measure of a household’s socio-economic status and is used as a proxy for a household’s income. It is made up of five categories (quintiles). The five categories are: ‘poorest’ (coded as 0), ‘poorer’ (coded as 1); ‘middle’ (coded as 2); ‘richer’ (coded as 3); ‘richest’ (coded as 4).
Place of residence	Place of residence was of two types: ‘rural’ (coded as 1) and ‘urban’ (coded as 0)
Exposure to family planning messages through mass media	Five questions were asked whether they have a) heard about family planning on the radio in the last few months, b) heard about family planning on TV in the last few months, c) read about family planning in newspapers/magazines in the last few months, d) heard about family planning on internet and e) heard about family planning on wall painting and hoarding. Women were considered ‘exposed’ (coded as 0) to mass media if they had exposure to any of these sources; and as ‘not-exposed’ (coded as 1) if otherwise
Total number of children before the index child	The number of children born before the index child was categorized as: ‘One child’ (coded as 0) and ‘Two or more children’ (coded as 1)
Survival status of the previous child	The survival status of the previous child was of two types- ‘Dead’ (coded as 1) and ‘Alive’ (coded as 0)
Sex of the child before the index child	The gender/sex of the previous child was of two types- ‘Male’ (coded as 0) and ‘Female’ (coded as 1)
Desired number of sons	The number of male children/sons/boys responded wished to have over her life. The variable has two categories- ‘None’ (coded as 0) and ‘One or more sons’ (coded as 1)
Level of poverty in community	The proportion of poor mothers (those from the poorest and poorer quintiles) in the primary sampling unit. It was categorised as- ‘Low’ (< 33%)’ (coded as 0), ‘Medium’ (33–66%) (coded as 1), and ‘High’ (> 66%) (coded as 2).
Level of education in community	The average number of years of schooling in the primary sampling unit. It was categorised into ‘Low’ (up to 5 years)’ (coded as 0), ‘Medium’ (6–12 years) (coded as 1), and ‘High’ (> 12 years) (coded as 2).
Proportion of women with exposure on family planning through mass media in PSU	The proportion of mothers exposed to family planning messages through mass media in the primary sampling unit was categorised as ‘Low’ (< 33%)’ (coded as 0), ‘Medium’ (33–66%) (coded as 1), and ‘High’ (> 66%) (coded as 2).
Region of residence	The states of India were grouped into six regions: ‘Northern’ (coded as 1, consists of Jammu & Kashmir, Ladakh, Himanchal Pradesh, Punjab, Haryana, Chandigarh, Delhi, and Rajasthan), ‘Central’ (coded as 2, consists of Uttar Pradesh, Chhattisgarh, and Madhya Pradesh), ‘Eastern’ (coded as 3, consists of Bihar, Jharkhand, West Bengal, and Odisha), ‘Northeastern’ (coded as 4, consists of Sikkim, Assam, Arunachal Pradesh, Nagaland, Meghalaya, Manipur, Mizoram, and Tripura), ‘Western’ (coded as 5, consist of Maharashtra, Gujarat, Goa, and Dadra & Nagar Haveli and Daman & Diu), ‘Southern’ (coded as 6, consist of Lakshadweep, Puducherry, Andaman & Nicobar Islands, Kerala, Tamil Nadu, Karnataka, Andhra Pradesh, and Telangana).

## Results

### Prevalence of SBI in India and its states

**Fig. 2:**
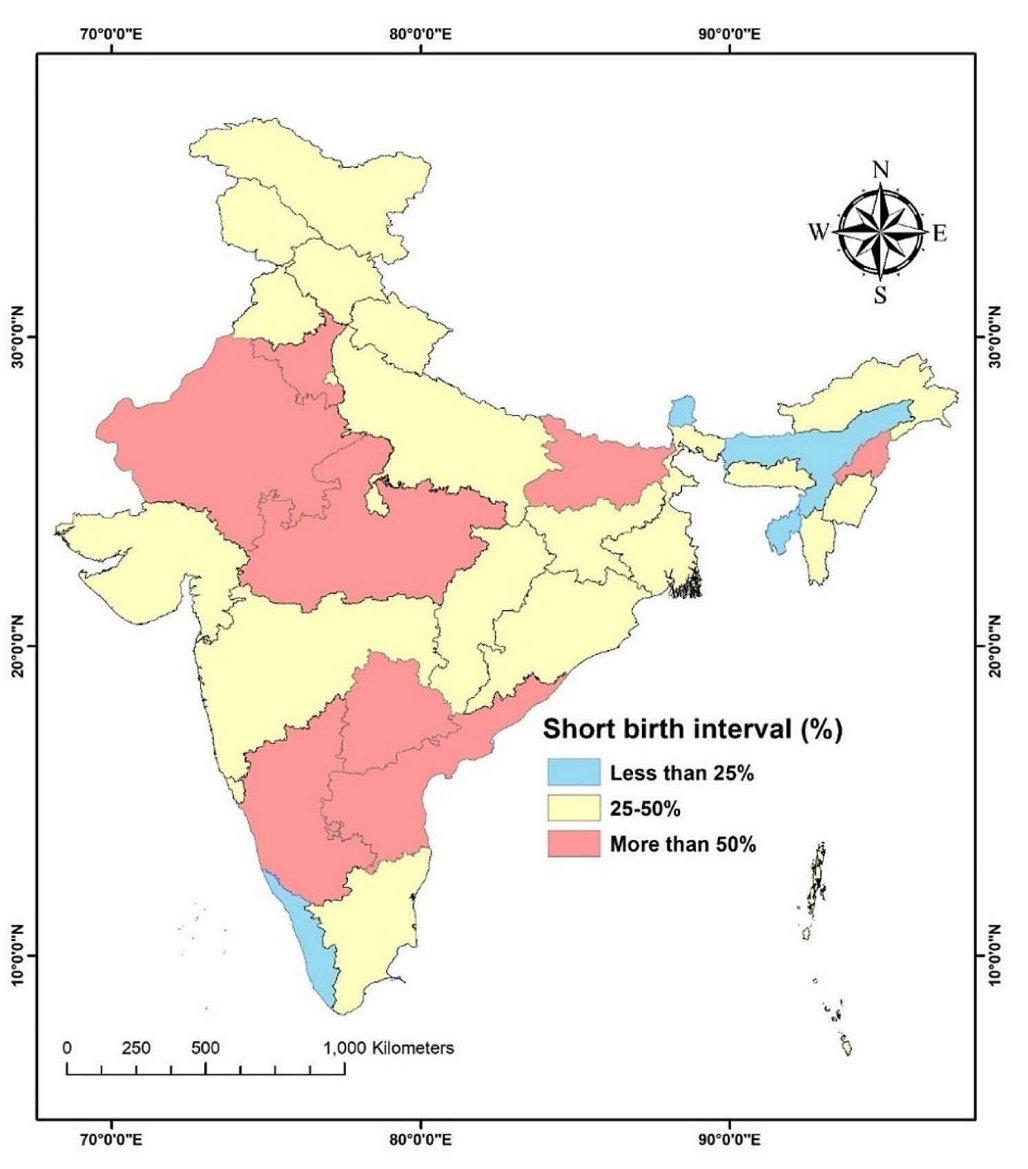
Proportion of women aged 15–49 years reporting short birth intervals in India, NFHS-5, 2019–21

The prevalence of SBI in India in 2019-21 was 47.8% (95% CI: 47.4–48.3). The prevalence varied considerably across the states of India (see Fig. 2). The prevalence of SBI was more than 50% in the states such as Bihar (61.2%, 60.2–62.4), Andhra Pradesh (60.6%, 57.8–63.4), Telangana (56.2%, 54.0-58.4), Nagaland (55.7%, 52.6–58.7), Madhya Pradesh (55.6%, 54.3–57.1), Rajasthan (51.4%, 49.9–53.0), Haryana (50.0%, 48.1–52.0), and Karnataka (51.6%, 49.3–53.9). On the other hand, the prevalence was less than 25% in the states of Sikkim (18.1%, 12.4–25.8), Tripura (18.3%, 15.4–21.7), Assam (20.9%, 19.1–22.9), and Kerala (21.5%, 19.1–24.2).

### Wealth-based disparity in SBI in India and its states

**Fig. 3:**
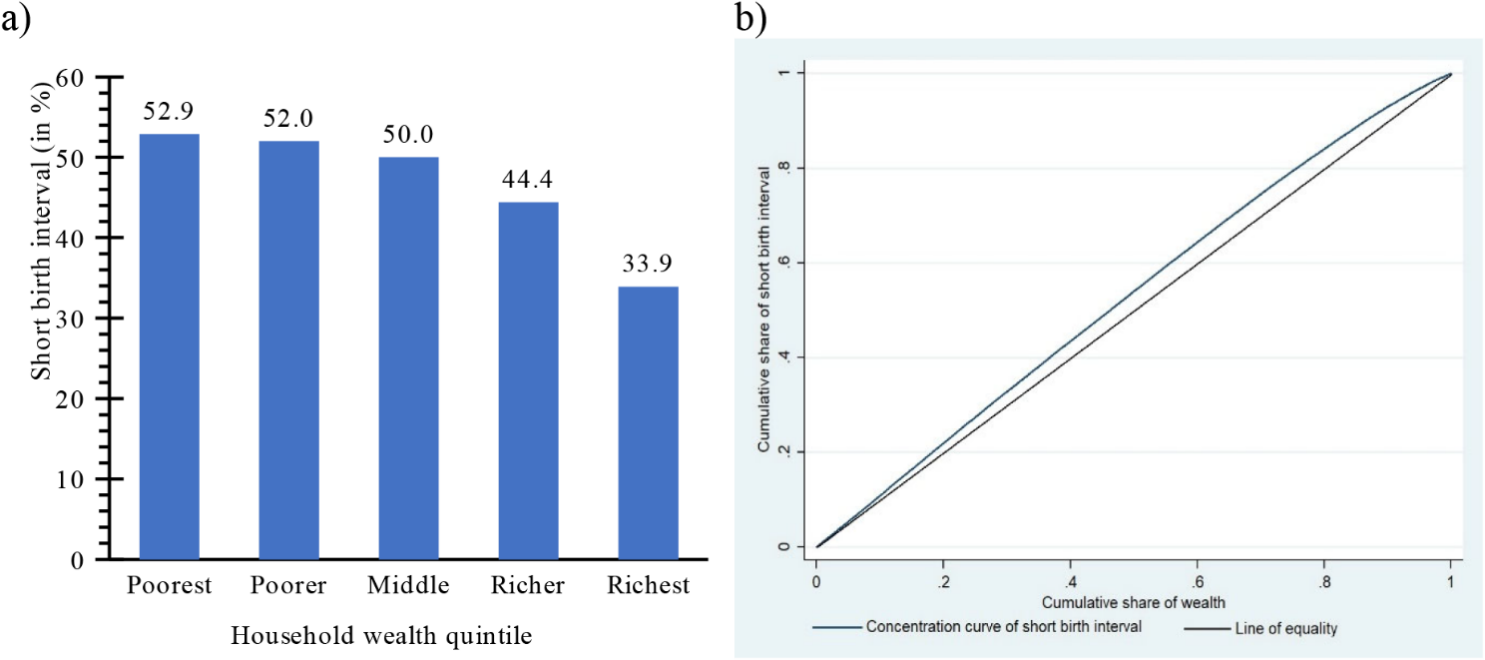
Wealth-based inequality in SBI in India, 2019-21

The prevalence of SBI in India decreases with an increase in the household wealth (see Fig. 3a). Approximately 53% of women in the poorest quintile reported SBI in India, whereas only 34% of women in the richest quintile reported SBI. This disparity was captured by concentration curve (CC) and ECI as well (see Fig. 3b). The CC depicts the association between the cumulative proportion of women reporting SBI on the vertical axis ranked by the cumulative percentage of the population ranked by the household wealth on the horizontal axis. The line of equality is represented by a 45-degree line, which indicates that the ECI is zero. The CC for the SBI lies above the line of equality, demonstrating that SBI was concentrated among the women from poorer households. The ECI was − 0.13 (Standard Error: 0.0035 and p-value < 0.001) and confirmed that there is wealth-based inequality in SBI in India.

Further, we calculated ECI for each state and UT individually to examine the variation in wealth-based inequality in SBI across the Indian states and UTs (see Table 2). The investigation of state ECIs revealed pro-poor inequality in the majority of the states. Among the states, the magnitude of pro-poor inequality in SBI was highest in Gujarat (ECI= -0.28, *p* < 0.001). Besides Gujarat, inequality in SBI was high (>-0.20) in Punjab (ECI= -0.28, *p* < 0.001), Manipur (ECI= -0.28, *p* < 0.001), Goa (ECI= -0.25, *p* < 0.018), Maharashtra (ECI= -0.23, *p* < 0.001), Madhya Pradesh (ECI= -0.21, *p* < 0.001), and Uttarakhand (ECI= -0.20, *p* < 0.001) as well. On the other hand, the lowest inequality in SBI was observed in the states of Kerala (ECI= -0.01, *p* = 0.643), Andhra Pradesh (ECI=-0.01, *p* = 0.820), Telangana (ECI= -0.05, *p* = 0.017) and Jharkhand (ECI= -0.04, *p* = 0.020).

**Table 2:** State-wise Erreygers Normalized Concentration Index, NFHS-5, 2019-21

States	Number of observations	ECI	Standard error	*p*-value
Andaman & Nicobar Islands	189	-0.333	0.071	< 0.001
Gujarat	4540	-0.282	0.017	< 0.001
Punjab	2616	-0.278	0.021	< 0.001
Manipur	1654	-0.275	0.025	< 0.001
Goa	96	-0.252	0.105	0.018
Maharashtra	4291	-0.226	0.017	< 0.001
DDDNH	369	-0.218	0.059	< 0.001
Madhya Pradesh	7658	-0.206	0.013	< 0.001
Uttarakhand	1824	-0.200	0.026	< 0.001
Delhi	1382	-0.194	0.030	< 0.001
Himachal Pradesh	1241	-0.189	0.032	< 0.001
Ladakh	194	-0.185	0.073	0.012
West Bengal	1874	-0.179	0.024	< 0.001
Assam	3151	-0.177	0.016	< 0.001
Odisha	4231	-0.177	0.015	< 0.001
Tamil Nadu	3062	-0.176	0.020	< 0.001
Karnataka	3811	-0.171	0.018	< 0.001
Haryana	3345	-0.166	0.020	< 0.001
Rajasthan	6826	-0.16	0.014	< 0.001
Meghalaya	3266	-0.149	0.020	< 0.001
Tripura	768	-0.142	0.032	< 0.001
Chandigarh	99	-0.136	0.111	0.223
Arunachal Pradesh	2705	-0.121	0.021	< 0.001
Nagaland	1485	-0.092	0.030	0.002
Jammu & Kashmir	2398	-0.077	0.023	0.001
Sikkim	270	-0.070	0.054	0.201
Uttar Pradesh	18,263	-0.069	0.009	< 0.001
Telangana	3564	-0.046	0.019	0.017
Mizoram	1343	-0.045	0.031	0.141
Bihar	10,379	-0.044	0.011	< 0.001
Jharkhand	5061	-0.038	0.016	0.020
Kerala	1349	-0.012	0.026	0.643
Andhra Pradesh	1412	-0.007	0.030	0.820
Lakshadweep	123	0.000	0.083	0.998
Chhattisgarh	4222	0.020	0.018	0.268
Puducherry	378	0.022	0.059	0.715
**India**	**109,439**	**-0.133**	**0.003**	**< 0.001**

*Note:* DDDNH: Daman & Diu and Dadra & Nagar Haveli; ECI: Erreygers Normalised Concentration Index

**Fig. 4:**
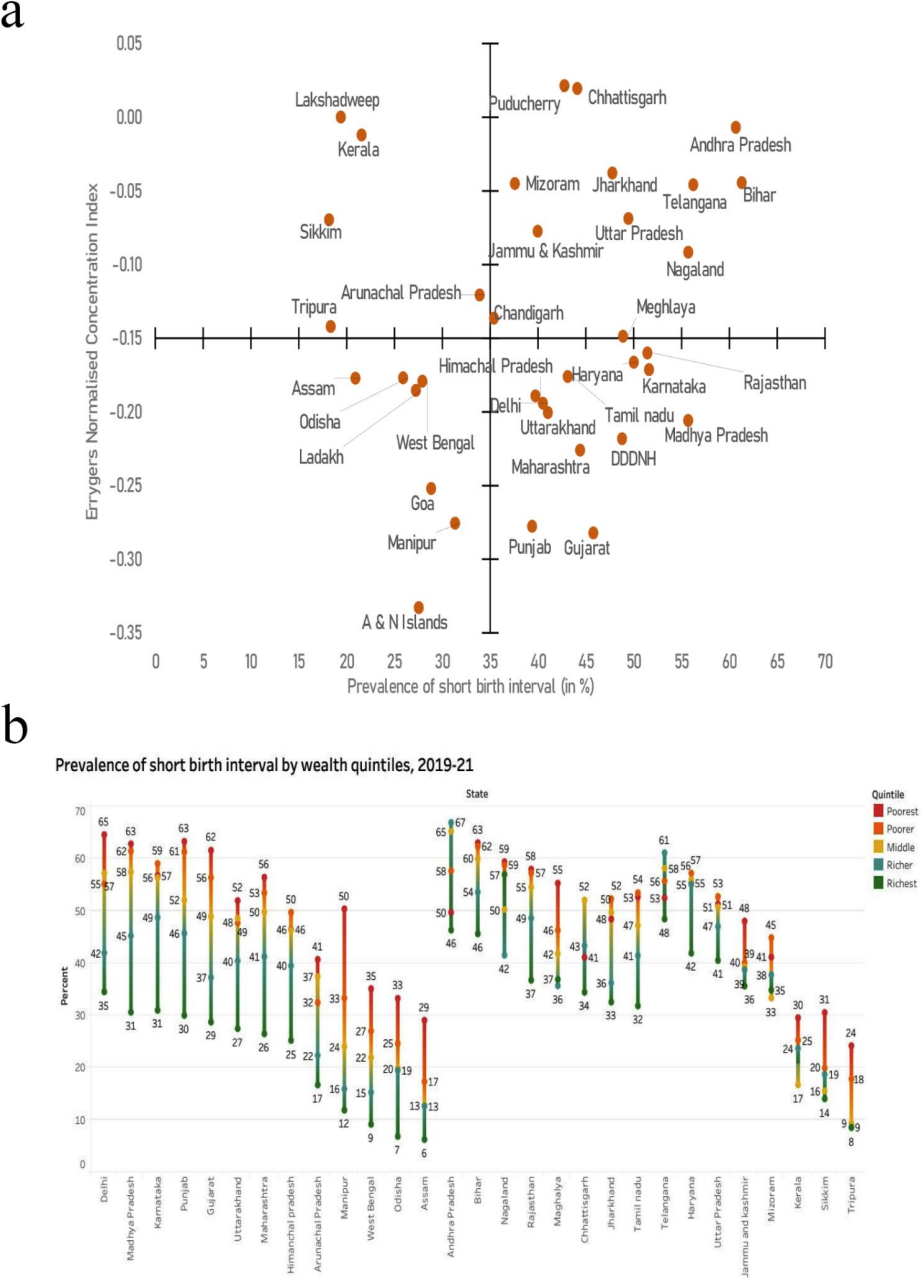
(**a**) Prevalence and wealth-based inequality in SBI across states of India, 2019-21. Note: A & N Islands: Andaman and Nicobar Islands; DDDNH: Daman and Diu and Dadra and Nagar Haveli. (**b**) Prevalence of SBI by wealth quintiles in selected states of India, 2019-21

Figure 4a illustrates the distribution of states based on their prevalence of SBI and the magnitude of wealth-based inequality in SBI, categorized into four quadrants. Meanwhile, Fig. 4b illustrates SBI prevalence by wealth quintiles in each state (for estimates and their confidence interval, see Appendix Table A3). In the lower right quadrant states are the states with high prevalence of SBI (more than 35.0%) and high wealth-based inequality in SBI (ECI  >− 0.15). This group of states includes the states of Himachal Pradesh (prevalence: 39.7%, ECI: -0.19), Uttarakhand (41.0%, -0.20), Punjab (39.4%, -0.28), Haryana (50.0%, -0.17), Rajasthan (51.4%, -0.16), Delhi (40.5%, -0.19), Madhya Pradesh (55.7%, -0.21), Gujarat (45.7%, -0.28), Maharashtra (44.4%, -0.23) in the northern and western regions, Karnataka (51.6%, -0.17) and Tamil Nadu (43.1%, -0.18) from the southern region, and Meghalaya (48.9%, -0.15) from the northeastern region. In contrast, states in the upper right quadrant which are Jammu and Kashmir (40%, -0.08), Uttar Pradesh (49.4%, -0.07), Bihar (61.3%, -0.04), Jharkhand (47.8%, -0.04), Telangana (56.2%, -0.05), Andhra Pradesh (60.7%, -0.01), and Nagaland (55.7%, -0.09) have high SBI prevalence but low wealth-based inequality in SBI. This is primarily due to a narrower gap in SBI between the rich and the poor in these states, as observed in Fig. 4b. Meanwhile, states in the lower left quadrant, including Ladakh (27.2%, -0.18), Odisha (25.9%, -0.18), West Bengal (27.9%, -0.18), Assam (20.9%, -0.18), Manipur (31.3%, -0.28), and Goa (28.8%, -0.25), demonstrate relatively low prevalence but high wealth-based inequality in SBI. This discrepancy is visually evident in Fig. 4b as well.

### Results of multilevel binary logistic regression

The initial stage of the multilevel model analysis involved evaluating whether our data supported the need to examine random effects at both the community and district levels. The Table 3 shows the result of the empty model. There was considerable variation in SBI across districts and communities. According to the VPC value obtained from the empty model, 12.7% and 9.0% of the total variance in SBI was attributed to the variation across communities and districts, respectively. For the sample characteristics and the prevalence of SBI by the background characteristics, see appendix Table A4 and A5.

**Table 3:** Parameter coefficient for the multilevel model for short birth interval - empty model without covariates

Random effects		Standard error
Community (PSU) random variance	0.088	0.009
Community (PSU) VPC (%)	12.70%	
District random variance	0.334	0.020
District VPC (%)	8.90%	

*Note:* VPC = Variance Partition Coefficient

The final model consisted of individual, community and district-level variables, and the result of the final model is shown in Table 4. The variance in the SBI was reduced to 6.0% across both the community and district. The result of the multilevel model revealed that women aged 15–24 (OR: 12.01, *p* < 0.001) and 25–34 (OR: 2.92, *p* < 0.001) were more likely to experience SBI compared to the women aged 35–49. Women who married between the ages of 20–25 had increased odds of experiencing SBI (OR: 1.78, *p* < 0.001), while those who married after the age of 25 had even higher odds (OR: 3.17, *p* < 0.001), in contrast to those who married before the age of 20. Women from poorest and poorer wealth quintiles were 1.97 and 1.73 times more likely to experience SBI as compared to women from the richest wealth quintile. SC, ST, and OBC women had slightly higher odds of experiencing SBI than those categorised as ‘Other’. Similarly, Muslim women (OR: 1.07, *p* = 0.008) and women from religions categorised as ‘Other’ (OR: 1.11, *p* = 0.003) had slightly higher odds of SBI than Hindu women.

The odds of SBI were 1.5 times higher among women who had two or more children before the index child (OR: 1.54, *p* < 0.001) compared to women who had only one child before the index child. The odds of SBI were twice higher (OR: 2.35, *p* = 0.036) among women whose previous child was no more. Women who were not exposed to family planning messages through mass media were slightly more likely to experience SBI (OR: 1.05, *p* = 0.008) compared to those women who were exposed. The likelihood of SBI was slightly higher among women residing in rural areas (OR: 1.07, *p* = 0.002) compared to those in urban areas. Furthermore, women living in communities with a higher proportion of poor individuals exhibited increased odds of SBI, with those in high-poverty areas having an 8% higher likelihood (OR: 1.08, *p* = 0.004), and those in medium-poverty areas having a 6% higher likelihood (OR: 1.06, *p* = 0.014), compared to women in low-poverty communities.

Women living in the communities with low (OR: 1.18, *p* < 0.001) and medium (OR: 1.14, *p* < 0.001) average years of schooling were 18% and 14% more likely to experience SBI than those women who lived in communities with higher average years of schooling, respectively. The odds of experiencing SBI were 6% higher among women with a medium level of exposure to family planning messages through mass media (OR: 1.06, *p* = 0.002) than women in a community with a high level of exposure. Women residing in the Northeastern (OR: 0.44, *p* < 0.001) and Eastern (OR: 0.67, *p* < 0.001) regions were 56% and 33% less likely to experience SBI than women residing in the Northern region [see Table 4].

**Table 4:** Results of multilevel logistic regression

Variable	Odds Ratio	S. E.	*p*-value	95% CI	
				Lower CI	Upper CI
**Age (in years)**					
15–24	12.01	0.36	< 0.001	11.32	12.75
25–34	2.92	0.07	< 0.001	2.79	3.05
35–49	**ref**				
**Age at marriage**					
Less than 20	**ref**				
20–25	1.78	0.03	< 0.001	1.73	1.84
More than 25	3.17	0.11	< 0.001	2.97	3.39
**Level of education**					
No education	**ref**				
Primary	1.02	0.02	0.370	0.98	1.07
Secondary	1.05	0.02	0.010	1.01	1.10
Higher	0.99	0.03	0.849	0.93	1.06
**Social Group**					
SC	1.18	0.03	< 0.001	1.13	1.24
ST	1.14	0.03	< 0.001	1.07	1.21
OBC	1.12	0.02	< 0.001	1.07	1.17
Others	**ref**				
**Religion**					
Hindu	**ref**				
Muslim	1.07	0.03	0.008	1.02	1.12
Others	1.11	0.04	0.003	1.04	1.19
**Wealth quintile**					
Poorest	1.97	0.07	< 0.001	1.85	2.11
Poorer	1.73	0.06	< 0.001	1.63	1.84
Middle	1.62	0.05	< 0.001	1.53	1.72
Richer	1.39	0.04	< 0.001	1.32	1.47
Richest	**ref**				
**Exposure to family planning messages**					
Yes	**ref**				
No	1.05	0.02	0.008	1.01	1.09
**No. of children before index child**					
1	**ref**				
2 or more	1.54	0.02	< 0.001	1.49	1.59
**Sex of previous child**					
Male	**ref**				
Female	1.03	0.01	0.037	1.00	1.06
**Desired number of sons**					
None	**ref**				
1 or more	1.05	0.02	0.036	1.00	1.10
**Survival status of last child**					
Alive	**ref**				
Dead	2.35	0.08	< 0.001	2.21	2.51
**Community level variable**					
**Place of residence**					
Urban	**ref**				
Rural	1.07	0.02	0.002	1.02	1.11
**Level of poverty in PSU**					
Low	**ref**				
Medium	1.06	0.03	0.014	1.01	1.11
High	1.08	0.03	0.004	1.03	1.14
**Level of education in PSU**					
Low	1.18	0.04	< 0.001	1.10	1.27
Medium	1.14	0.04	< 0.001	1.07	1.22
High	**ref**				
**Proportion of women with exposure on family planning through mass media in PSU**					
Low	1.00	0.03	0.866	0.95	1.05
Medium	1.06	0.02	0.002	1.02	1.11
High	**ref**				
**District level variables**					
**Region of residence**					
Northern	**ref**				
Central	1.09	0.06	0.136	0.97	1.23
Eastern	0.67	0.04	< 0.001	0.59	0.76
Northeastern	0.44	0.03	< 0.001	0.38	0.50
Western	0.96	0.07	0.528	0.83	1.10
Southern	1.07	0.07	0.301	0.94	1.21
**Random effects**					
Community (PSU) random variance	0.015	0.00		0.01	0.02
Community (PSU) VPC (%)	6.0				
District random variance	0.212	0.01		0.19	0.24
District VPC (%)	6.0				

*Note:* S.E: Standard Error, CI: Confidence Interval, ref: Reference category, SC: Scheduled Caste, ST: Scheduled Tribe, OBC: Other Backward Classes, PSU: Primary Sampling Unit, and VPC: Variance Partition Coefficient

## Discussion

The study aimed to investigate wealth-based disparities in SBI and to identify factors associated with SBI at various levels—individual, community, and district—in India. The findings revealed that approximately half of mothers in India experienced SBI, with a concentration of SBI among women from poorer households. Moreover, significant variation in wealth-based inequality in SBI was observed across Indian states, with states such as Gujarat, Maharashtra, Madhya Pradesh, Goa, Punjab, Uttarakhand, and Manipur exhibiting higher levels of inequality. Several factors emerged as statistically significant associates of SBI in India. These factors included age, age at marriage, wealth quintile, total number of children before the index child, and survival status of previous children.

Our results indicate that SBI is associated with household wealth. This finding is consistent with earlier research [11, 13, 26, 30, 44, 45]. This finding may be attributed to the improvement in access to health education, family planning resources, and maternal healthcare services as women’s economic status improves. SBI disproportionately affect women from poorer backgrounds. This could be due to intertwined relationship between health and poverty, as poorer communities bear a heavier burden of ill health. Socioeconomic disparities hinder access to crucial health information and family planning resources to women, limiting their autonomy in reproductive decisions. This intensifies health inequalities, potentially compromising the well-being of both women and their children. Furthermore, a recent study indicated that higher socioeconomic status correlates with greater utilization of modern contraceptives, leading to a decreased likelihood of subsequent childbirth and thereby extending the interval between successive births [13, 26]. Mothers from economically disadvantaged households face significant barriers in accessing and utilizing effective contraception methods, increasing their vulnerability to SBI. Limited knowledge and financial constraints obstruct their ability to utilize modern and emergency contraceptive options, worsening the impact of economic disparities on reproductive health outcomes [15, 46, 47].

The prevalence of SBI decreased with the increase in woman’s age. Several previous studies have found a similar result; younger women are more likely to have a SBI when compared to older women [13, 19, 28]. This could be because women of younger age have motives to have their desired number of children quickly, whereas women of older age have a concern about infertility or menopause; in addition to that, older women have more autonomy and decision-making power about their reproductive choices [3, 6, 13].

The study found that women who married for the first time between the ages of 20–24 and 25–29 had higher probabilities of experiencing SBI between births compared to those who married before turning 20. Previous research on this topic has produced similar findings [48, 49]. This trend may be attributed to the fact that women who marry at an older age tend to want to complete their families more rapidly. Women marrying in their early to late twenties might be more conscious of their biological clocks and the potential risks associated with delayed childbearing, prompting them to expedite the process of having children [8, 19, 48].

Caste emerged as a significant predictor of SBI, and this result aligns with previous studies that have found that mothers from SC exhibits a higher likelihood of experiencing SBI [50, 51]. This could be because women from this social group are characterized by lower socioeconomic status, limited education, and lack of awareness about reproductive health and optimal birth interval [13].

Religion emerged as another significant factor associated with SBI, with Muslim women and women belonging to other religions slightly more likely to experience SBI. This finding aligns with previous research [26, 45, 51, 52] and the higher likelihood may be attributed to the underutilization of contraceptives and birth control methods among the followers of Muslim faith and other religions [53]. Additionally, factors such as low women’s autonomy and lack of awareness could contribute to the slightly elevated levels of SBI among the women of these faiths.

The study indicates that mothers with a birth order of two or more are more likely to experience SBI, which contradicts previous research [11, 28, 53]. One possible explanation for this discrepancy could that couples with higher birth orders may be intentionally seeking to have a larger family, which could lead them to have children in quick succession. Another reason could be a lack of knowledge and access to modern contraceptive methods, making it difficult for these couples to effectively plan and space their pregnancies. Additionally, these couples may not be fully aware of the potential disadvantages associated with having reduced intervals between births.

Mortality of a preceding child was associated with increased likelihood of SBI. This phenomenon may be attributed to the halt of breastfeeding following the death of the previous child, potentially leading to an earlier resumption of menstruation and ovulation among women. Consequently, some couples may unintentionally conceive another child within a reduced timeframe. Conversely, certain couples might intentionally seek to conceive another child expeditiously following the loss of the previous one. This finding aligns with earlier studies [8, 13, 19, 22, 31, 54].

Women exposed to mass media about family planning were slightly less likely to have SBI than those women without mass media exposure. The finding is similar to those of the existing literature. A reason for this finding could be that mass media plays an important role in creating awareness about small family size norms and family planning services, and women exposed to any type of mass media have a better understanding of the adverse effect the SBI can have on the health of both mother and child [5, 8, 11, 15, 22].

The study found place of residence a significant associate of SBI among women of reproductive age. Women residing in rural areas were more likely to experience SBI than their urban counterparts. The existing literature has also argued that despite government efforts, the rural residents still do not have easy access to modern healthcare services, exposure to information, and family planning methods [2, 5, 13, 15, 24, 29, 43]. This could one of the many reasons why women in rural areas still have a higher likelihood of experiencing SBI.

In 2017, the Government of India has launched ‘Mission Parivar Vikas’ to expand the accessibility of contraceptives and family planning services in the 146 high fertility districts. These districts are in the seven high-focus and high total fertility rates of Uttar Pradesh, Madhya Pradesh, Bihar, Rajasthan, Chhattisgarh, Jharkhand, and Assam. Under the initiative, there is a scheme of using ASHAs services for counselling newlywed couples to ensure a gap of two years in birth after the marriage and those couples with already one child to have an interval of three years after the birth of the first child. In addition to this, information, education and communication materials are used to raise awareness about the importance of ensuring appropriate space between childbirths [55, 56]. Under the scheme, ASHA is paid up to 1000 rupees in the form of incentives per couple. Every state except Kerala, Tamil Nadu, and Goa has approved the scheme’s spacing component.

Despite the government’s ongoing efforts and emphasis on family planning, the prevalence of SBI in India remains significantly higher than in many developing countries. Given the profound implications of SBI for maternal and child health, it is imperative for the government to intensify its efforts to reduce its prevalence. The observation that SBI varies considerably across states underscores the need for targeted interventions in specific geographical areas. Recognizing this variability, the government can tailor its strategies to address the unique challenges and socio-cultural factors influencing reproductive behaviours in different regions. Moreover, certain states exhibit both high levels of SBI and pronounced wealth-based inequalities in SBI. In these states, concerted efforts are required not only to reduce the overall proportion of women experiencing SBI but also to narrow the gap in SBI prevalence between socioeconomic groups. This necessitates a multifaceted approach that addresses both structural inequalities and barriers to accessing family planning services among marginalized populations. To effectively tackle the issue of higher prevalence of SBI and its associated health risks, the government should prioritize initiatives aimed at improving access to contraceptive services, promoting awareness about the importance of birth spacing, and addressing socioeconomic disparities that hinder equitable access to reproductive healthcare.

The key strengths of this study are as follows: it uses the latest nationally representative and population-based survey data to measure wealth-based inequality in SBI in India and its states. In the study, along with television, radio, and newspapers/magazines, we have included internet and hoardings while calculating the variable representing exposure for family planning messages through mass media. We also acknowledge that the study has a few limitations. First, we omitted a variable pertaining to the duration of breastfeeding before the index child as it had missing observations. Secondly, the absence of data on exact gestational periods in the NFHS-5 survey led us to assume an average gestational duration of nine months for all births. Third, we considered the birth interval of the most recent child only because the information about the previous child, which was needed in the analysis, could not be obtained due to data limitations. Additionally, the exclusion of missing observations in certain variables utilized for analysis was necessary to maintain the integrity of the study; however, it may have introduced bias into the results. Therefore, caution is advised when interpreting the findings.

## Conclusion

The significant wealth-based inequality in SBI in India underscores the urgency of implementing targeted interventions that prioritize economically disadvantaged women, particularly in regions with high SBI prevalence. In addressing this disparity, special attention should be directed towards younger mothers and those belonging to socially disadvantaged groups. These vulnerable populations often face additional barriers to accessing reproductive healthcare services and may be at higher risk of adverse maternal and child health outcomes. Implementing targeted interventions tailored to the specific needs and circumstances of these groups is essential for achieving equitable access to reproductive healthcare services and ensuring the well-being of mothers and their children.

### Electronic supplementary material

Below is the link to the electronic supplementary material.

Supplementary Material 1

## Data Availability

The dataset analyzed during the current study is available in the Demographic and Health Surveys (DHS) repository, https://dhsprogram.com/data/available-datasets.cfm, and can be obtained for free by sending an online request. Codes used in the analysis can be made available from the corresponding author on request.
