# Supplementary material for: Wealth-based disparities in the prevalence of short birth interval in India: insights from NFHS-5
Source: Popul Health Metr. 2024 Jul 11;22:14. doi: 10.1186/s12963-024-00334-0 (PMC11238510; doi:10.1186/s12963-024-00334-0)

**Appendix files**

**Table A1: Background characteristics of missing and non-missing observations.**

| **Variable** | **Non-missing observations**  **(%, 95% CI)** | | **Missing observations**  **(%, 95% CI)** | |
| --- | --- | --- | --- | --- |
| **Age group (in years)** |  |  |  |  |
| 15–24 | 19.8 | [19.4,20.1] | 22.4 | [20.9,23.9] |
| 25–34 | 67.7 | [67.3,68.1] | 63.9 | [62.3,65.5] |
| 35–49 | 12.6 | [12.3,12.8] | 13.7 | [12.6,14.9] |
| **Age at marriage** |  |  |  |  |
| Less than 20 years | 59.8 | [59.3,60.3] | 63.7 | [61.8,65.6] |
| 20–25 years | 35.5 | [35.1,36.0] | 30.3 | [28.6,32.1] |
| Above 25 years | 4.7 | [04.5,04.9] | 6.0 | [05.1,06.9] |
| **Level of education** |  |  |  |  |
| No education | 25.2 | [24.7,25.7] | 22.0 | [20.4,23.6] |
| Primary | 13.7 | [13.4,14.0] | 17.7 | [16.3,19.2] |
| Secondary | 48.6 | [48.1,49.1] | 51.5 | [49.6,53.4] |
| Higher | 12.4 | [12.0,12.8] | 8.8 | [07.7,10.0] |
| **Social group** |  |  |  |  |
| Schedule Caste | 24.7 | [24.2,25.2] | 11.1 | [09.4,13.0] |
| Schedule Tribe | 10.6 | [10.2,10.9] | 8.4 | [06.8,10.4] |
| Other Backward Class | 46.3 | [45.7,46.9] | 22.4 | [19.9,25.1] |
| Others | 18.4 | [17.9,19.0] | 9.5 | [07.8,11.5] |
| Don't know | 0.0 |  | 48.5 | [44.6,52.5] |
| **Religion** |  |  |  |  |
| Muslim | 81.1 | [80.5,81.7] | 43.1 | [40.7,45.6] |
| Hindu | 14.6 | [14.0,15.2] | 53.1 | [50.6,55.6] |
| Others | 4.3 | [04.1,04.6] | 3.7 | [03.1,04.5] |
| **Household wealth index** |  |  |  |  |
| Poorest | 25.7 | [25.1,26.3] | 31.6 | [29.7,33.6] |
| Poorer | 22.0 | [21.6,22.4] | 23.1 | [21.4,24.8] |
| Middle | 19.6 | [19.2,20.0] | 16.3 | [15.0,17.7] |
| Richer | 18.0 | [17.6,18.5] | 15.9 | [14.5,17.4] |
| Richest | 14.7 | [14.2,15.2] | 13.1 | [11.5,14.9] |
| **Place of residence** |  |  |  |  |
| Urban | 26.2 | [25.4,27.1] | 29.5 | [26.9,32.3] |
| Rural | 73.8 | [72.9,74.6] | 70.5 | [67.7,73.1] |
| **Exposure to family planning messages** | | |  |  |
| Yes | 72.3 | [71.8,72.8] | 65.4 | [63.4,67.4] |
| No | 27.7 | [27.1,28.2] | 34.6 | [32.6,36.6] |
| **Total number of children before index child** | | | |  |
| One child | 55.0 | [54.5,55.5] | 58.7 | [56.9,60.5] |
| Two or more children | 45.0 | [44.5,45.5] | 41.3 | [39.5,43.1] |
| **Survival status of previous child** |  |  |  |  |
| Alive | 95.3 | [95.1,95.4] | 95.4 | [94.6,96.1] |
| Dead | 4.7 | [04.6,04.9] | 4.6 | [03.9,05.4] |
| **Sex of the previous child** |  |  |  |  |
| Male | 47.5 | [47.1,47.9] | 49.2 | [47.4,50.9] |
| Female | 52.5 | [52.1,52.9] | 50.8 | [49.1,52.6] |
| **Desired number of sons** |  |  |  |  |
| Zero | 11.3 | [11.0,11.7] | 12.6 | [11.4,13.9] |
| One or more sons | 88.7 | [88.3,89.0] | 87.4 | [86.1,88.6] |

**Table A2: Variance inflation factor**

| **Variable** | **VIF** | **1/VIF** |
| --- | --- | --- |
| Wealth index | 1.53 | 0.66 |
| Place of residence | 1.26 | 0.79 |
| Number of children before the index child | 1.24 | 0.8 |
| Education | 1.23 | 0.81 |
| Age (in years) | 1.13 | 0.89 |
| Exposure to mass media | 1.11 | 0.9 |
| Age at marriage (in years) | 1.06 | 0.94 |
| Religion | 1.05 | 0.95 |
| Use of contraceptive | 1.04 | 0.96 |
| Sex of the previous child | 1.02 | 0.98 |
| Survival status of previous child | 1.01 | 0.99 |
| Preference for son | 1.01 | 0.99 |
| Mean VIF | 1.14 |  |

**Table A3: Prevalence of short birth interval by wealth quintiles, 2019-21**

| **State** | **n** | **SBI (in %)** | **Poorest*** | **Poorer*** | **Middle*** | **Richer*** | **Richest*** | **P-value** |
| --- | --- | --- | --- | --- | --- | --- | --- | --- |
| **Jammu and Kashmir** | 2,398 | 39.9, [37.3,42.6] | 48, [41.8,54.4] | 40.1, [35.1,45.4] | 39.3, [34.2,44.7] | 38.7, [34.1,43.5] | 35.6, [29.8,41.9] | 0.058 |
| **Himanchal Pradesh** | 1,241 | 39.7, [36.2,43.4] | 46.4, [33.8,59.5] | 49.7, [40.7,58.8] | 46.3, [39.7,53.1] | 39.5, [32.5,47.0] | 25.2, [19.3,32.2] | < 0.001 |
| **Punjab** | 2,616 | 39.3, [37.1,41.7] | 63.3, [47.3,76.9] | 61.3, [52.8,69.2] | 52, [45.9,58.2] | 45.7, [41.2,50.4] | 30, [27.1,33.1] | < 0.001 |
| **Chandigarh** | 99 | 35.3, [24.9,47.4] | 100 | 51.5, [05.4,95.2] | 37.4, [05.3,86.5] | 42.5, [28.4,58.1] | 31.1, [20.3,44.5] | 0.320 |
| **Uttarakhand** | 1,824 | 41.0, [37.8,44.3] | 51.9, [42.5,61.3] | 47.7, [41.4,54.1] | 48.6, [43.4,53.9] | 40.4, [33.9,47.4] | 27.4, [22.3,33.4] | < 0.001 |
| **Haryana** | 3,345 | 50.0, [48.1,52.0] | 55.3, [44.5,65.7] | 57.2, [52.1,62.2] | 56, [52.0,60.0] | 55.1, [51.5,58.6] | 41.9, [39.1,44.8] | < 0.001 |
| **Delhi** | 1,382 | 40.5, [37.2,43.9] | 64.6, [23.1,91.7] | 55.1, [43.3,66.5] | 57.2, [49.0,65.1] | 41.9, [35.3,49.0] | 34.5, [30.8,38.4] | < 0.001 |
| **Rajasthan** | 6,826 | 51.4, [49.9,53.0] | 58, [54.8,61.1] | 57.3, [54.6,59.9] | 54.6, [51.7,57.4] | 48.6, [45.1,52.2] | 36.7, [33.0,40.7] | < 0.001 |
| **Uttar Pradesh** | 18,263 | 49.4, [48.6,50.3] | 51.2, [49.8,52.8] | 52.7, [51.2,54.4] | 50.8, [48.9,52.7] | 47, [44.7,49.4] | 40.5, [38.0,43.2] | < 0.001 |
| **Bihar** | 10,379 | 61.2, [60.2,62.4] | 63, [61.5,64.6] | 62.4, [60.3,64.4] | 60, [57.1,62.9] | 53.6, [49.9,57.3] | 45.6, [38.1,53.4] | < 0.001 |
| **Sikkim** | 270 | 18.1, [12.4,25.8] | 30.5, [11.6,59.6] | 19.9, [09.9,36.0] | 15.5, [07.8,28.7] | 18.6, [08.2,37.2] | 14, [02.9,47.4] | 0.851 |
| **Arunachal Pradesh** | 2,705 | 33.9, [31.6,36.3] | 40.7, [36.1,45.5] | 32.4, [28.6,36.6] | 37.4, [33.0,42.2] | 22.3, [18.0,27.4] | 16.6, [10.1,26.3] | < 0.001 |
| **Nagaland** | 1,485 | 55.7, [52.6,58.7] | 59.4, [55.3,63.4] | 58.7, [52.7,64.6] | 50.3, [41.8,58.8] | 41.5, [32.6,51.1] | 57, [41.5,71.3] | 0.012 |
| **Manipur** | 1,654 | 31.3, [28.1,34.8] | 50.4, [45.0,55.8] | 33.3, [29.3,37.7] | 24, [18.9,30.1] | 15.8, [10.5,23.3] | 11.8, [05.4,24.1] | < 0.001 |
| **Mizoram** | 1,343 | 37.5, [34.4,40.9] | 41.1, [33.7,49.1] | 44.8, [38.5,51.4] | 33.3, [27.8,39.4] | 37.7, [31.3,44.8] | 34.8, [27.2,43.4] | 0.169 |
| **Tripura** | 768 | 18.3, [15.4,21.7] | 24.1, [19.6,29.5] | 17.8, [13.2,23.6] | 9.3, [05.4,15.7] | 8.4, [03.2,20.9] | 8.5, [01.0,45.1] | 0.001 |
| **Meghalaya** | 3,266 | 48.8, [46.2,51.6] | 55.3, [51.6,59.0] | 46.3, [42.2,50.5] | 41.8, [35.0,49.0] | 35.6, [25.7,47.1] | 36.9, [16.7,63.1] | 0.002 |
| **Assam** | 3,151 | 20.9, [19.1,22.9] | 29, [26.0,32.3] | 17.3, [14.9,20.1] | 12.8, [09.2,17.6] | 12.6, [08.1,19.2] | 6.2, [02.5,14.8] | < 0.001 |
| **West Bengal** | 1,874 | 27.9, [25.5,30.4] | 35.1, [31.6,38.8] | 26.9, [22.9,31.6] | 21.9, [16.9,28.0] | 15.2, [10.3,21.9] | 9.1, [03.2,23.3] | < 0.001 |
| **Jharkhand** | 5,061 | 47.7, [46.1,49.5] | 48.4, [46.3,50.6] | 52.3, [49.1,55.5] | 49.7, [44.8,54.7] | 36.2, [30.9,41.9] | 32.5, [25.6,40.4] | < 0.001 |
| **Odisha** | 4,231 | 25.9, [24.3,27.5] | 33.2, [30.9,35.7] | 24.6, [21.9,27.5] | 19.8, [16.7,23.5] | 19.4, [15.2,24.6] | 6.8, [04.0,11.4] | < 0.001 |
| **Chhattisgarh** | 4,222 | 44.1, [42.1,46.1] | 41.1, [38.3,44.1] | 45.7, [42.2,49.4] | 52.1, [47.5,56.7] | 43.3, [37.9,48.9] | 34.4, [27.9,41.6] | < 0.001 |
| **Madhya Pradesh** | 7,658 | 55.6, [54.3,57.1] | 62.8, [60.8,64.9] | 61.5, [58.9,64.1] | 57.5, [54.3,60.6] | 45.2, [41.6,49.0] | 30.6, [26.5,35.1] | < 0.001 |
| **Gujarat** | 4,540 | 45.7, [43.7,47.9] | 61.6, [57.6,65.5] | 56.4, [52.7,60.1] | 48.9, [44.8,53.1] | 37.2, [33.0,41.8] | 28.7, [23.9,34.1] | < 0.001 |
| **DDDNH** | 369 | 48.7, [43.2,54.4] | 49.5, [36.5,62.6] | 55.3, [44.3,66.0] | 59.6, [49.8,68.7] | 39.7, [28.4,52.3] | 23.4, [12.6,39.5] | 0.002 |
| **Maharashtra** | 4,291 | 44.4, [42.0,46.8] | 56.4, [51.3,61.5] | 53.4, [49.0,57.9] | 49.8, [45.5,54.2] | 41.2, [37.6,45.1] | 26.4, [21.1,32.7] | < 0.001 |
| **Andhra Pradesh** | 1,412 | 60.6, [57.8,63.4] | 49.7, [37.9,61.7] | 57.7, [52.4,62.8] | 65.2, [60.7,69.6] | 66.9, [61.7,71.8] | 46.3, [38.2,54.7] | < 0.001 |
| **Karnataka** | 3,811 | 51.6, [49.3,53.9] | 56.8, [52.1,61.4] | 59, [55.4,62.7] | 56.4, [52.8,60.0] | 48.7, [44.8,52.9] | 30.9, [24.7,38.1] | < 0.001 |
| **Goa** | 96 | 28.8, [20.8,38.4] | 100 | 24.7, [03.3,76.2] | 58.5, [29.2,82.8] | 31.8, [16.9,51.8] | 18.2, [09.0,33.6] | 0.057 |
| **Lakshadweep** | 123 | 19.3, [12.6,28.5] |  | 0 | 19.5, [07.1,43.7] | 19.2, [11.3,29.8] | 21.1, [08.9,42.3] | 0.837 |
| **Kerala** | 1,349 | 21.5, [19.1,24.2] | 29.5, [13.1,53.9] | 25.2, [15.2,39.0] | 16.7, [12.5,22.0] | 23.6, [19.8,28.1] | 20.8, [17.2,25.0] | 0.216 |
| **Tamil Nadu** | 3,062 | 43.1, [41.0,45.2] | 52.7, [42.8,62.4] | 53.5, [48.5,58.4] | 47.2, [43.2,51.3] | 41.4, [38.1,44.9] | 31.8, [26.5,37.7] | < 0.001 |
| **Puducherry** | 378 | 42.7, [35.2,50.7] | 17.6, [03.5,56.0] | 43.7, [23.1,66.9] | 43.1, [22.8,66.1] | 44, [33.2,55.5] | 42.7, [29.3,57.4] | 0.863 |
| **A & N Islands** | 189 | 27.5, [21.0,35.2] | 51.8, [29.6,73.4] | 46.3, [30.0,63.5] | 29.2, [14.2,50.8] | 18.7, [09.2,34.3] | 8.1, [01.9,28.6] | 0.009 |
| **Telangana** | 3,564 | 56.2, [54.0,58.4] | 52.5, [44.9,60.0] | 55.7, [51.5,59.9] | 58.2, [54.8,61.5] | 61.1, [56.9,65.3] | 48.4, [42.6,54.4] | 0.001 |
| **Ladakh** | 194 | 27.2, [20.0,35.9] | 40, [26.0,56.1] | 37.2, [24.9,51.4] | 12, [04.9,26.7] | 22.6, [10.7,41.7] | 37.9, [15.3,67.5] | 0.021 |
| **India** | 1,09,439 | 47.8, [47.4,48.3] | 52.9, [52.1,53.7] | 52, [51.2,52.9] | 50, [49.1,51.0] | 44.4, [43.4,45.4] | 33.8, [32.7,35.1] | < 0.001 |

Note: n = Total number of observations, %= weighted percentage of short birth interval, *= Percentage of short birth interval by wealth quintile along with 95% Confidence Interval, DDDNH = Daman & Diu and Dadra & Nagar Haveli, and A & N Islands = Andaman and Nicobar Islands.

**Table A4: Descriptive statistics of sampled women aged 15–49 years by background characteristics, NFHS-5, 2019–21**

| **Variables** | **n= (109,439)** | **Weighted %** |
| --- | --- | --- |
| **Age group (in years)** |  |  |
| 15–24 | 19,965 | 19.8 |
| 25–34 | 73,497 | 67.7 |
| 35–49 | 15,977 | 12.6 |
| **Age at marriage** |  |  |
| Less than 20 years | 62,337 | 59.8 |
| 20–25 years | 40,450 | 35.5 |
| Above 25 years | 6,652 | 4.7 |
| **Level of education** |  |  |
| No education | 28,043 | 25.2 |
| Primary | 15,734 | 13.7 |
| Secondary | 53,893 | 48.6 |
| Higher | 11,769 | 12.4 |
| **Social group** |  |  |
| Schedule Caste | 23,755 | 24.7 |
| Schedule Tribe | 23,818 | 10.6 |
| OBC | 44,092 | 46.3 |
| Others | 17,774 | 18.4 |
| **Religion** |  |  |
| Muslim | 81,758 | 14.6 |
| Hindu | 13,654 | 81.1 |
| Others | 14,027 | 4.3 |
| **Household wealth index** |  |  |
| Poorest | 31,118 | 25.7 |
| Poorer | 25,983 | 22.0 |
| Middle | 21,010 | 19.6 |
| Richer | 17,833 | 18.0 |
| Richest | 13,495 | 14.7 |
| **Place of residence** |  |  |
| Urban | 21,819 | 26.2 |
| Rural | 87,620 | 73.8 |
| **Exposure to family planning messages** | |  |
| Yes | 78,049 | 72.3 |
| No | 31,390 | 27.7 |
| **Total number of children before index child** | |  |
| One child | 58,115 | 55.0 |
| Two or more children | 51,324 | 45.0 |
| **Survival status of previous child** | |  |
| Alive | 1,04,204 | 95.3 |
| Dead | 5,235 | 4.7 |
| **Sex of the previous child** |  |  |
| Male | 52,238 | 47.5 |
| Female | 57,201 | 52.5 |
| **Desired number of sons** |  |  |
| Zero | 11,484 | 11.3 |
| One or more sons | 97,955 | 88.7 |
| **Level of poverty in community** | |  |
| Low | 42,086 | 43.2 |
| Medium | 17,854 | 16.5 |
| High | 49,499 | 40.3 |
| **Level of exposure to FP messages in community** | |  |
| Low | 17,683 | 15.6 |
| Medium | 20,983 | 18.8 |
| High | 70,773 | 65.7 |
| **Level of women’s education in community** | |  |
| Low | 36,753 | 32.6 |
| Medium | 64,825 | 58.8 |
| High | 7,861 | 8.6 |
| **Region of residence** |  |  |
| Northern | 19,925 | 13.5 |
| Central | 30,143 | 30.1 |
| Eastern | 21,545 | 25.1 |
| Northeastern | 14,642 | 2.6 |
| Western | 9,296 | 12.0 |
| Southern | 13,888 | 16.8 |
| **India** | 1,09,439 | 100 |
| Note: n- Total sample, FP- Family Planning, SC-Scheduled Caste, ST-Scheduled Tribe, OBC-Other Backward Class, %-Weighted percentage | | |

**Table A5: Prevalence of women having short birth interval by background characteristics, NFHS-5, 2019–21**

| **Background characteristics** | **Short birth interval (%)** | **95% CI** | **Chi^2^** | ***p*-value** |
| --- | --- | --- | --- | --- |
| **Age group (in years)** |  |  | 7698.09 | < 0.001 |
| 15–24 | 71.8 | [70.9,72.6] |  |  |
| 25–34 | 44.7 | [44.2,45.2] |  |  |
| 35–49 | 26.8 | [25.8,27.8] |  |  |
| **Age at marriage** |  |  | 154.36 | < 0.001 |
| Less than 20 | 49.2 | [48.6,49.7] |  |  |
| 20–25 | 46.4 | [45.6,47.1] |  |  |
| More than 25 | 41.8 | [39.9,43.7] |  |  |
| **Level of education** |  |  | 562.08 | < 0.001 |
| No education | 50.7 | [49.9,51.5] |  |  |
| Primary | 49.2 | [48.1,50.3] |  |  |
| Secondary | 48.3 | [47.6,48.9] |  |  |
| Higher | 38.7 | [37.4,39.9] |  |  |
| **Social Group** |  |  | 507.88 | < 0.001 |
| SC | 51.0 | [50.1,51.8] |  |  |
| ST | 49.2 | [48.1,50.4] |  |  |
| OBC | 48.5 | [47.9,49.2] |  |  |
| Others | 41.0 | [39.9,42.1] |  |  |
| **Religion** |  |  | 77.28 | < 0.001 |
| Hindu | 48.4 | [47.9,48.9] |  |  |
| Muslim | 46.2 | [45.0,47.5] |  |  |
| Others | 42.7 | [40.9,44.5] |  |  |
| **Wealth quintile** |  |  | 1849.87 | < 0.001 |
| Poorest | 52.9 | [52.1,53.7] |  |  |
| Poorer | 52.1 | [51.2,52.9] |  |  |
| Middle | 50.0 | [49.1,51.0] |  |  |
| Richer | 44.4 | [43.4,45.4] |  |  |
| Richest | 33.9 | [32.7,35.1] |  |  |
| **Exposure to family planning messages** | |  | 273.02 | < 0.001 |
| Yes | 46.3 | [45.7,46.8] |  |  |
| No | 51.9 | [51.1,52.6] |  |  |
| **No. of children before index child** | |  | 131.85 | < 0.001 |
| 1 | 46.3 | [45.6,46.9] |  |  |
| 2 or more | 49.7 | [49.1,50.4] |  |  |
| **Sex of previous child** |  |  | 0.59 | 0.572 |
| Male | 47.9 | [47.3,48.6] |  |  |
| Female | 47.7 | [47.1,48.3] |  |  |
| **Desired number of sons** |  |  | 79.25 | < 0.001 |
| None | 44.1 | [42.8,45.4] |  |  |
| 1 or more | 48.3 | [47.8,48.8] |  |  |
| **Survival status of last child** |  |  | 963.12 | < 0.001 |
| Alive | 46.8 | [46.3,47.2] |  |  |
| Dead | 68.9 | [67.2,70.5] |  |  |
| **Place of residence** |  |  | 923.35 | < 0.001 |
| Urban | 40.1 | [39.1,41.1] |  |  |
| Rural | 50.6 | [50.1,51.1] |  |  |
| **Level of poverty in community** | |  | 881.97 | < 0.001 |
| Low | 42.7 | [41.9,43.4] |  |  |
| Medium | 51.3 | [50.3,52.3] |  |  |
| High | 51.9 | [51.2,52.6] |  |  |
| **Level of exposure to FP messages in community** | |  | 475.52 | < 0.001 |
| Low | 52.6 | [51.4,53.7] |  |  |
| Medium | 52.2 | [51.2,53.2] |  |  |
| High | 45.4 | [44.9,46.0] |  |  |
| **Level of women education in community** | |  | 1074.23 | < 0.001 |
| Low | 52.6 | [51.9,53.3] |  |  |
| Medium | 47.2 | [46.6,47.8] |  |  |
| High | 33.9 | [32.3,35.5] |  |  |
| **Region of residence** |  |  | 577.62 | < 0.001 |
| Northern | 47.0 | [46.0,47.9] |  |  |
| Central | 50.3 | [49.6,51.0] |  |  |
| Eastern | 48.7 | [47.7,49.7] |  |  |
| Northeastern | 28.1 | [26.8,29.4] |  |  |
| Western | 44.8 | [43.1,46.6] |  |  |
| Southern | 48.0 | [46.8,49.1] |  |  |
| **India** | 47.8 | [47.4,48.3] |  |  |

Note: FP: Family Planning, SC: Scheduled Caste, ST: Scheduled Tribe, OBC: Other Backward class, %: Weighted percentage, and CI: Confidence Interval

**Figure A1: State-wise wealth-based inequality in the short birth interval materials among women aged 15–49 years in India, NFHS-5, 2019–21**


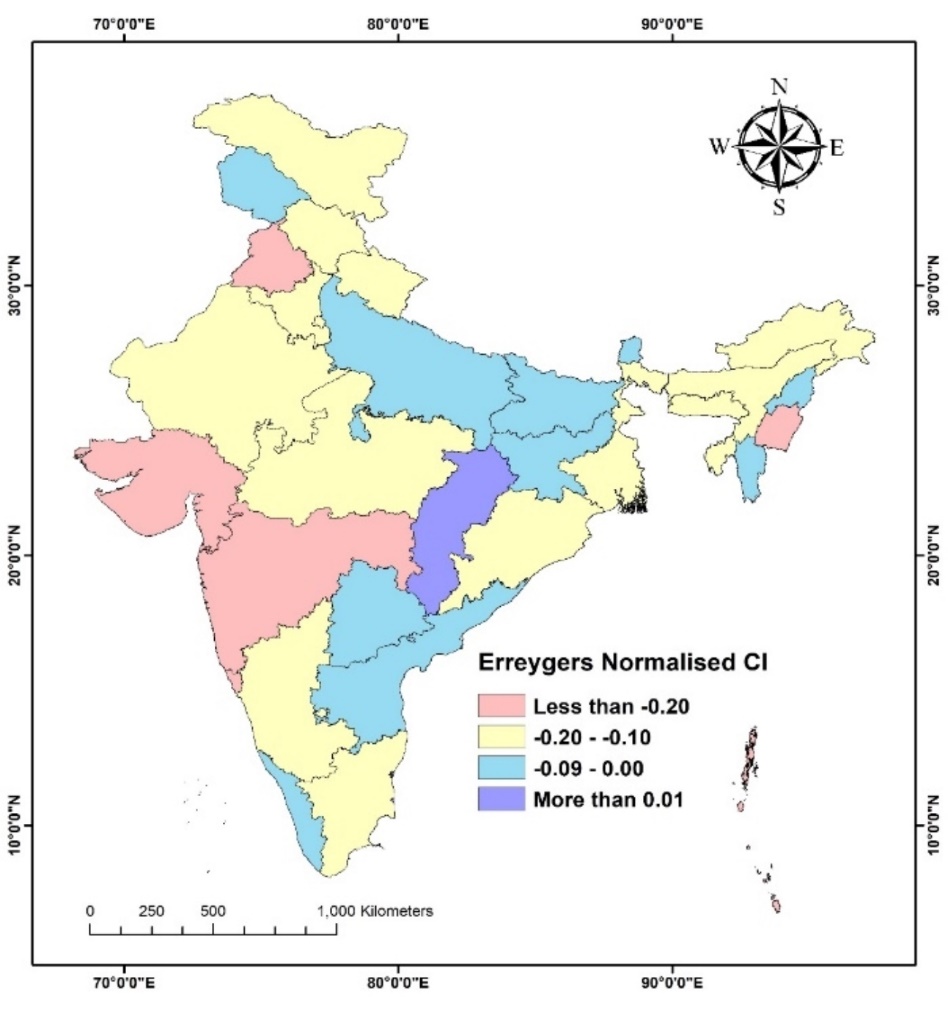

Supplement: Supplementary file 1 — Supplementary Material 1 [file 12963_2024_334_MOESM1_ESM.docx]
